# Socket preservation and its impact on final prosthetic outcomes

**DOI:** 10.6026/973206300221860

**Published:** 2026-03-31

**Authors:** Sidharth Shrikant Joshi, Himanshi Kalra, Gomati Dhande, Deepak R, Prateek Tripathy, Arup Ratan Das

**Affiliations:** 1 Department of Periodontology, Yogita Dental College and Hospital, Khed, Ratnagiri, India; 2 Department of Prosthodontics, Geetanjali Dental College, Udaipur, Rajasthan, India; 3 Department of Dentistry, Shri Jawahir Hospital, Jaisalmer, Rajasthan, India; 4 Department of Prosthodontics and Crown and Bridge, Vinayaka Mission's Sankarachariyar Dental College and Hospital, Salem, India; 5 Department of Oral and Maxillofacial surgery, Hi-Tech Dental College and Hospital, Bhubaneswar, India

**Keywords:** Socket preservation, alveolar ridge preservation, prosthetic outcomes, bone graft, dental implant feasibility, ridge resorption, esthetic rehabilitation, interdisciplinary dentistry, oral surgery, prosthodontics

## Abstract

Post-extraction alveolar bone resorption remains a significant clinical challenge, often compromising implant placement and aesthetic outcomes. Hence, fifty patients undergoing tooth extraction were equally divided into two groups: one group received socket preservation using bone grafts and membranes, while the other underwent conventional healing. Clinical, radiographic, and prosthetic parameters were evaluated over an 18-month period. The socket preservation group demonstrated fewer post-operative complications, along with significantly improved aesthetic scores and higher patient satisfaction. Thus, we show the importance of bone grafting procedures in enhancing clinical efficiency, prosthetic outcomes, predictability and aesthetics following tooth extraction.

## Background:

A common dental procedure called tooth extraction starts a chain reaction of remodelling of both soft and hard tissues, which frequently results in significant changes to the dimensions of the alveolar ridge. The majority of post-extraction ridge loss, primarily in the horizontal and vertical planes, happens within the first three months, as shown by Araújo and Lindhe [1]. This can cause problems with future implant placement and prosthetic results. The significance of successful ridge preservation techniques is highlighted by Horváth *et al.* systematic review, which confirmed that the extent of ridge reduction can vary, based on a number of factors, including the surgical technique and the location of the extracted tooth [2]. One of the main strategies for reducing alveolar bone loss has been the post-extraction use of graft materials. In their comparison of extraction alone and xenograft application, Barone *et al.* found that grafted sockets preserved ridge dimensions considerably better [3]. In a similar vein, Naenni pointed out that although ridge volume is consistently lost during natural healing, ridge preservation methods can prevent and restrict this resorption, particularly in the anterior aesthetic zones [4]. By measuring the beneficial effects of ridge preservation on bone volume retention, a meta-analysis by Avila-Ortiz *et al.* supported these findings and came to the conclusion that preserved sockets show noticeably less horizontal and vertical bone loss than conventionally healed sites [5]. Site and associates also highlighted the stability of ridge dimensions when grafting is used; demonstrating long-term advantages even after the prosthetic phase is finished [6].

In a clinical study employing a collagen membrane and freeze-dried bone allograft, Isabella *et al.* showed that the collagen membrane not only preserved ridge dimensions but also produced favourable soft tissue healing profiles, which are crucial for aesthetic results [7]. Although they observed differences in integration times and soft tissue compatibility, Mardas *et al.* compared xenografts with synthetic alternatives and found that they were equally effective in preserving ridge volume [8]. When Chappuis *et al.* compared post-extraction changes with and without preservation; they found that the preservation group had better emergence profiles, better ridge integrity and less need for secondary augmentation [9]. When combined with a membrane, mineralised bone allografts, according to Wang *et al.* offered structural support that allowed for early implant placement, making subsequent restorative procedures easier [10].

Practical surgical guidelines for successful ridge preservation were provided by a thorough review by Narby *et al.* which emphasised the significance of flap management, graft selection and traumatic extraction in guaranteeing the best results [11]. Araújo and colleagues, in a separate study, explored the natural healing patterns of extraction sockets and concluded that despite complete epithelialization, significant bone resorption persists without grafting [12]. In comparison to ungrafted sockets, Botticelli *et al.* experimentally confirmed that socket grafting considerably lowers hard tissue loss and promotes higher bone quality for implant integration [13]. Furthermore, the biological timeline of socket healing was documented by Fan *et al.* demonstrating how early graft placement may be able to intercept and favourably influence healing outcomes [14]. Study by Darby *et al.* [15] reported that significant dimensional changes occur in the alveolar ridge following tooth extraction, particularly during the early healing phase. The authors emphasized that ridge preservation techniques can effectively minimize horizontal and vertical bone loss. Therefore, it is of interest to compare the socket preservation with traditional healing and assessing clinical, radiographic, aesthetic and patient-reported outcomes over an 18-month period in this study.

## Methodology:

The Departments of Oral and Maxillofacial Surgery and Prosthodontics at a tertiary dental teaching institution collaborated to conduct this prospective observational clinical study over the course of 18 months, from January 2023 to June 2024. The purpose of the study was to evaluate how socket preservation affected the upkeep of the alveolar ridge and the results of prosthetics after tooth extraction. Fifty patients between the ages of 20 and 65 who needed their non-restorable teeth extracted and then had to wait for prosthetic rehabilitation were enrolled. Systemically healthy people (ASA I or II), proper oral hygiene and intact post-extraction socket walls were requirements for inclusion. Individuals with a history of head and neck radiation therapy, ongoing periodontal infections, tobacco use, or systemic conditions that impair healing were not included. Two groups of 25 participants each were randomly selected. After atraumatic extractions, either engraft or demineralised freeze-dried bone allograft (DFDBA) was inserted into the socket in Group A (socket preservation group). A restorable collagen membrane was also applied in certain cases. Either primary or secondary intention healing was used to achieve closure, depending on the clinical circumstances. Atraumatic extractions were performed on Group B (conventional healing group) without the use of grafts or membranes and the sockets were left to heal on their own. Trained oral surgeons followed established protocols to perform all surgical procedures under local anaesthesia. Both groups received the same postoperative instructions and prescriptions. At one week, one month, three months and six months after surgery, intraoral photos and periapical radiographs or CBCT scans were used for clinical and radiographic evaluations.

All patients proceeded to the prosthodontic phase following a minimum of three months of healing. Assessments were conducted on ridge form, soft tissue health and suitability for implants and other fixed or removable prostheses. Experienced prosthodontists used standard clinical protocols to fabricate final prostheses that were customised to the patient's condition and prosthetic requirements. The complexity of prosthetic rehabilitation, clinical and radiographic graft integration (in Group A) and horizontal and vertical ridge dimensional changes were the main outcome measures. Secondary outcomes included patient-reported satisfaction (using a 10-point VAS scale), functional stability, infection, graft failure and prosthetic redesign, as well as prosthetic success as determined by aesthetics (Pink and White Aesthetic Scores). Structured case sheets were used to document the entire data and SPSS version 25.0 was used for analysis. For continuous variables, ANOVA and independent t-tests were employed; for categorical data, chi-square tests were used. Statistical significance was defined as a p-value < 0.05. To find predictors of the best prosthetic results, multivariate regression analysis was used. All participants provided written informed consent and ethical clearance (IRB No: XYZ/2023/067).

## Results:

For the study, 50 patients (26 men and 24 women) between the ages of 20 and 65 were split equally into two groups: Group A (socket preservation) and Group B (conventional healing), each with 25 participants. There were no significant baseline differences between the groups in terms of age, gender, or distribution of extraction sites and all patients finished the clinical follow-up. In general, the healing process after surgery went smoothly. One patient in Group A experienced mild membrane exposure and two more experienced mild postoperative discomfort; all of these issues resolved on their own without the need for additional care. Infection or graft failure was not observed in either group. Group A demonstrated noticeably better results in terms of alveolar ridge preservation at the 3-month follow-up. The mean horizontal ridge loss was 1.2 ± 0.4 mm in Group A and 3.8 ± 0.6 mm in Group B (p < 0.001), as shown in Table 1 (see PDF). Additionally, there was a p-value of less than 0.001 for vertical ridge loss in the preservation group (0.9 ± 0.3 mm) as opposed to the conventional healing group (2.7 ± 0.5 mm). In 96% of Group A sockets, radiographic graft integration was seen. 88% of patients in Group A and 52% of patients in Group B had preserved papillary (p = 0.005). 80% of Group A received an excellent rating for soft tissue contour, which is significantly higher than Group B's 36% rating (p = 0.003). Significant variations were also noted during the prosthetic phase Table 2 (see PDF). The need for additional augmentation was significantly lower in Group A (8%) compared to Group B (52%) and implant placement feasibility was higher in Group A (88%) than in Group B (60%) with p = 0.01 (p < 0.001). In terms of aesthetic evaluation, Group A had higher Pink Aesthetic Scores (11.2 ± 1.3) than Group B (8.4 ± 1.6) and Group A had better White Aesthetic Scores (8.9 ± 0.7) than Group B (7.1 ± 1.1), both of which had p < 0.01. Group A's average patient satisfaction, as determined by a 10-point VAS, was 9.2 ± 0.6, while Group B's was 7.4 ± 1.0 (p = 0.002). Although this difference was not statistically significant, Group A's implant success rate was 100%, while Group B's was 92%. Overall, both tables' results support the idea that, when compared to conventional healing, socket preservation produces noticeably better results in terms of ridge dimensions, soft tissue health, prosthetic planning and patient-centered success.

## Discussion:

The study's findings unequivocally highlight how crucial socket preservation is to preserving the volume of the alveolar ridge following extraction and improving prosthetic results. When examined in an interdisciplinary setting with both prosthodontists and oral surgeons, this effect is particularly noticeable. The increasing clinical literature consensus that prompt intervention following tooth extraction reduces alveolar bone remodelling and collapse is supported by the notable dimensional preservation seen in Group A as opposed to Group B. Reduced horizontal and vertical ridge loss in the socket preservation group was one of the study's most clinically significant findings. This is in line with Ten Heggeler *et al.* [16], who showed in a thorough review that post-extraction resorption, was greatly reduced by the use of barrier membranes and bone substitutes. According to their research, ridge preservation techniques improve implant site quality and reduce the need for additional bone grafting operations. In our study, 88% of patients in Group A were prepared for direct implant placement, while only 60% of patients in Group B were. This suggests a more favourable clinical pathway with fewer surgical procedures. With socket preservation, soft tissue results also significantly improved. Collagen-based materials used in socket preservation promote soft tissue revascularisation and contour stability, both of which are essential for preserving gingival papillae and the aesthetic architecture of anterior zones, according to Lim *et al.* [17]. This was supported by our data, which showed that Group A patients had better emergence profiles and more soft tissue fill, two characteristics that are crucial for long-term aesthetic success, particularly when it comes to implant-supported restorations. Kim and Ku [18] histological analysis lends more credence to these findings by showing that xenografts, like deproteinised bovine bone, aid in soft tissue adaptation in addition to providing structural scaffolding for new bone growth. The results of our study, which used comparable materials and procedures, were similar to those of Maiorana *et al.* [19], who assessed collagen membranes in combination with grafts derived from pigs and reported stable ridge dimensions as well as cosmetically pleasing outcomes for implant therapy. From the perspective of prosthodontics, socket preservation enhances prosthetic design, function and aesthetic predictability in addition to facilitating structural retention. According to Roccuzzo *et al.* [20], socket preservation enhances overall prosthesis fit and aesthetics cuts down on the number of surgical and prosthetic adjustments needed and streamlines treatment planning. Group A patients in our study had more harmonious tissue contours and needed substantially fewer prosthetic adjustments. However, because of ridge deficiencies, Group B often needed compensatory design changes like flanged pontics or ridge-lap designs. Additionally, Fickl *et al.* [21] highlighted the problems with unpreserved sockets, pointing out that spontaneous healing frequently results in ridge concavity, which makes maintaining prosthetic hygiene and aesthetics more difficult. These restrictions were noticeable in our Group B cohort, where compromises in prosthetics were often required, lowering patient satisfaction and aesthetic appeal. Barone *et al.* [22] provided additional confirmation of the long-term advantages of socket preservation, stating that preserved sites showed better results in delayed implant placements and more consistent ridge dimensions. Similar patterns were seen in our investigation, where Group A demonstrated improved soft tissue integration at follow-up in addition to higher implant success rates. Additional information was provided by Temmerman *et al.* [23], who demonstrated that ridge preservation dramatically lowers the risk of gingival recession and peri-implant bone loss over time. Our Group A patients had fewer of these long-term issues, which emphasises how crucial socket preservation is during the surgical and prosthetic stages of treatment. Additionally, patient-reported results supported the preservation of the socket. According to our data, patients in Group A reported higher levels of satisfaction with treatment effectiveness, hygiene maintenance and aesthetics. While natural healing can be biologically complete, Schropp *et al.* [24] showed that it frequently leads to unpredictable and excessive ridge reduction, jeopardising restorative plans. This was in line with the results from our Group B, where ridge collapse necessitated secondary surgical or prosthetic interventions for almost half of the patients. Lastly, this study emphasises the importance of working across disciplines. Better decisions about flap designs, material selection and prosthetic planning were made possible by the close collaboration between the prosthodontic and surgical teams. This team-based approach is similar to contemporary dental paradigms, which show that integrated care produces better functional and aesthetic results. In summary, socket preservation is a patient-centred, clinically validated strategy that improves implant planning reduces tissue loss following extraction and increases prosthetic predictability. It promotes long-term success and increased patient satisfaction when carried out using an interdisciplinary model. A recent study published in the Journal of Advances in Oral Health [25] also supports these findings, concluding that socket preservation techniques significantly enhance clinical outcomes by reducing bone resorption and improving both functional and aesthetic results following tooth extraction.

## Conclusion:

Socket preservation significantly reduces alveolar ridge resorption and improves both soft tissue profiles and implant feasibility. This leads to enhanced esthetic outcomes, greater patient satisfaction and fewer prosthetic complications. Thus, an interdisciplinary approach between oral surgeons and prosthodontists ensures optimal, predictable rehabilitation results.
